# *Uncoupling protein 2* haplotype does not affect human brain structure and function in a sample of community-dwelling older adults

**DOI:** 10.1371/journal.pone.0181392

**Published:** 2017-08-03

**Authors:** Verena Heise, Enikő Zsoldos, Sana Suri, Claire Sexton, Anya Topiwala, Nicola Filippini, Abda Mahmood, Charlotte L. Allan, Archana Singh-Manoux, Mika Kivimäki, Clare E. Mackay, Klaus P. Ebmeier

**Affiliations:** 1 OHBA, Oxford Centre for Human Brain Activity, University of Oxford, Oxford, United Kingdom; 2 Department of Psychiatry, University of Oxford, Oxford, United Kingdom; 3 Nuffield Department of Population Health, University of Oxford, Oxford, United Kingdom; 4 Northumberland, Tyne and Wear NHS Foundation Trust and Institute of Neuroscience, Newcastle University, Newcastle upon Tyne, United Kingdom; 5 Department of Epidemiology and Public Health, University College London, London, United Kingdom; 6 Centre for Research in Epidemiology and Population Health, Hôpital Paul Brousse, INSERM, U1018, Villejuif, France; 7 Oxford Health NHS Foundation Trust, Warneford Hospital, Oxford, United Kingdom; Boston Children's Hospital / Harvard Medical School, UNITED STATES

## Abstract

Uncoupling protein 2 (UCP2) is a mitochondrial membrane protein that plays a role in uncoupling electron transport from adenosine triphosphate (ATP) formation. Polymorphisms of the *UCP2* gene in humans affect protein expression and function and have been linked to survival into old age. Since UCP2 is expressed in several brain regions, we investigated in this study whether *UCP2* polymorphisms might 1) affect occurrence of neurodegenerative or mental health disorders and 2) affect measures of brain structure and function. We used structural magnetic resonance imaging (MRI), diffusion-weighted MRI and resting-state functional MRI in the neuroimaging sub-study of the Whitehall II cohort. Data from 536 individuals aged 60 to 83 years were analyzed. No association of *UCP2* polymorphisms with the occurrence of neurodegenerative disorders or grey and white matter structure or resting-state functional connectivity was observed. However, there was a significant effect on occurrence of mood disorders in men with the minor alleles of -866G>A (rs659366) and Ala55Val (rs660339)) being associated with increasing odds of lifetime occurrence of mood disorders in a dose dependent manner. This result was not accompanied by effects of *UCP2* polymorphisms on brain structure and function, which might either indicate that the sample investigated here was too small and underpowered to find any significant effects, or that potential effects of *UCP2* polymorphisms on the brain are too subtle to be picked up by any of the neuroimaging measures used.

## Introduction

Uncoupling protein (UCP) 2 is a proton carrier found in the inner mitochondrial membrane. It is responsible for uncoupling electron transport through the electron transfer chain from the active pumping of protons across the inner mitochondrial membrane, thus uncoupling electron transport from adenosine triphosphate (ATP) formation [1]. UCP2 is abundantly expressed in brain tissue and in non-human primates it can be found in areas, such as the hypothalamic nuclei, pituitary gland, brainstem (incl. raphe nucleus and locus coeruleus), ventral tegmental area and substantia nigra [2]. There are few investigations of UCP2 expression in the human brain but expression has been shown in the cortex and basal ganglia with increased expression in the periphery of ischemic stroke lesions [3]. It affects neuronal function in multiple ways, e.g. as a regulator of production of reactive oxygen species (ROS) [4]. Thus UCP2 provides a plausible link between mitochondrial (dys-)function, ROS production, ageing and neurodegeneration [5]. For example, UCP2 overexpression in mice protects neurons in the substantia nigra of a Parkinson’s disease model [6]. UCP2 also appears to play a role in neuronal plasticity and regeneration [7], and human UCP2 expression extends the lifespan of flies and mice [8].

There are several polymorphisms of the *UCP2* gene in humans. The most frequently investigated polymorphisms are the -866G>A promoter polymorphism, which affects *UCP2* mRNA expression, and the Ala55Val polymorphism in the exon of the gene leading to a substitution of amino acids, which affects the degree of uncoupling and thus protein function [4]. Some studies have shown links between these polymorphisms and risk of obesity, insulin resistance and diabetes [9], although there are many conflicting reports on these effects [10]. A study that investigated the effects of *UCP* gene variants on longevity found that *UCP2* polymorphisms affect survival into old age [11].

Due to the potential link between *UCP2* gene variants, ROS production and longevity, we investigated in this study, whether *UCP2* polymorphisms affect the risk of developing neurodegenerative diseases and whether they account for any variance in brain structure or function.

We examined data from the Whitehall II imaging sub-study. Whitehall II is an ongoing epidemiological study that has followed-up an initial cohort of 10,000 British civil servants since 1985, with the aim of advancing knowledge of the causal chain through which social circumstances influence health [12]. Over the past 30 years a wealth of social, behavioural and biological data have been acquired, which makes Whitehall II a unique study of ageing. Within the Whitehall II imaging sub-study brain magnetic resonance imaging (MRI) scans of 800 participants randomly selected from the original Whitehall II study of 10,308 British civil servants were acquired [13]. In the study presented here, data from 563 individuals aged 60 to 83 years that have been scanned prior to January 2016 were analyzed.

We investigated effects of *UCP2* polymorphisms on risk of neurodegenerative disease (defined as having a clinical diagnosis of Parkinson’s disease, Alzheimer’s disease, mild cognitive impairment (MCI), stroke, transient ischemic attack or multiple sclerosis) and the risk of having a lifetime diagnosis of mental health disorder (defined as current or past episodes of mood disorders or anxiety disorders) in this sample. To determine effects of *UCP2* polymorphisms on brain structure and function, we investigated grey and white matter structure, as well as functional brain connectivity at rest, using structural, diffusion and functional MRI.

## Material and methods

### Participants

In order to make the Whitehall II neuroimaging sub-sample as representative as possible of the cohort at baseline, a random sample of 800 Whitehall II Phase 11 participants willing and able to give informed consent were invited to attend the imaging sub-study at the Oxford Centre for Functional MRI of the Brain (FMRIB). To achieve a sufficient number of participants with depression, we enriched the sample by 30 participants with depressive symptoms based on previous Whitehall II clinical examinations. We excluded participants with contraindications to MRI scanning (including but not limited to a history of claustrophobia, certain metallic implants and metallic injury to the eye), or who were unable to travel to Oxford without assistance. Data from 563 individuals aged 60 to 83 years that have been scanned prior to January 2016 were included in the analyses.

Ethical approval for the Whitehall II study was obtained from the University College London Medical School committee on the ethics of human research and all participants provided written informed consent. Ethical approval for neuroimaging data acquisition was granted generically for the “Protocol for non-invasive magnetic resonance investigations in healthy volunteers” (MSD/IDREC/2010/P17.2) by the University of Oxford Central University/ Medical Science Division Interdisciplinary Research Ethics Committee (CUREC/ MSD-IDREC), who also approved the specific protocol: “Predicting MRI abnormalities with longitudinal data of the Whitehall II sub-study” (MSD-IDREC-C1-2011-71, PI: KPE). The Health Research Authority NRES Committee South Central—Oxford C approved the study: “Biological mechanisms and risk factors in late onset depression” (REC Reference: 10/H0606/71, PI: CLA) that allowed us to examine participants with a past history of depressive symptoms.

The study follows the Medical Research Council (MRC) Policy on data sharing, i.e. images and other data will be available for analysis by other groups after completion of the study, as is the case with the Whitehall II study (see http://www.ucl.ac.uk/whitehallII/data-sharing).

### Clinical and cognitive assessment

Each participant recruited for the Whitehall II imaging sub-study underwent a detailed clinical and cognitive assessment lasting up to two hours. The clinical assessment consisted of a (A) self-administered questionnaire, a (B) semi-structured clinical interview and (C) cognitive assessment. For this study only a subset of the data was used, which is described here in more detail. For an overview of all assessments, please see [13].

- General Health Questionnaire-30 (GHQ-30 [14]): The GHQ-30 is a 30-item self-administered screening questionnaire for the detection of psychiatric illness in non-psychiatric clinical and community settings.
- Montreal Cognitive Assessment (MoCA [15]): The MoCA is a 30-point cognitive screening test assessing multiple cognitive domains: visuo-spatial abilities, short-term memory recall, executive function, attention, orientation and working memory, language and orientation to time and space.
- Centre for Epidemiological Studies Depression Scale (CES-D [16]): The CES-D is short self-report scale that measures major depressive symptomatology in the general population.
- Test of Premorbid Functioning (TOPF [17]): The TOPF consists of a list of seventy written words, which must be read aloud and is marked according to pronunciation. The TOPF is used to estimate an individual's level of intellectual functioning before the onset of injury or illness. Premorbid IQ can be calculated from the raw score, adjusted for sex and years of education.
- Structured Clinical Interview for DSM-IV-TR Axis I Disorders [18]: The SCID-I is a semi-structured interview for diagnosing current and past DSM-IV Axis I disorders and is administered by a trained graduate psychologist or psychiatrist.

### Genotyping

DNA was extracted from whole blood samples (via magnetic bead technology; Medical Solutions, Nottingham, UK) and normalized to a concentration of 50 ng/μl. Participants were genotyped for two polymorphisms of the *UCP*2 gene, -866G>A (rs659366) and Ala55Val (rs660339) using SNPLex (Applied Biosciences, Inc., Warrington, United Kingdom) by Medical Solutions Plc (Nottingham, United Kingdom)[19].

### Neuroimaging data acquisition

A detailed description of the image acquisition protocols and pre-processing steps for neuroimaging data can be found in [13]. In brief, scanning was carried out at FMRIB using a 3 T Siemens Magnetom Verio (Erlangen, Germany) Scanner with a 32-channel receive head coil. The neuroimaging protocol comprised both structural and functional sequences and lasted approximately 50 minutes. The MRI sequences that were analyzed for this study included high-resolution T1-weighted MRI (multi-echo MPRAGE [20], TR = 2530 ms, TE = 1.79/3.65/5.51/7.37 ms, flip angle = 7°, field of view = 256 mm, voxel dimension = 1 mm isotropic, acquisition time = 6 min 12 s), diffusion MRI (dMRI) (TR = 8900 ms, TE = 91.2 ms, field of view = 192 mm, voxel dimension = 2 mm isotropic, B-value = 1500 s/mm^2^, gradients applied = 60 isotropically distributed diffusion-weighted directions with b-value = 1500 s/mm^2^, 5 non-diffusion weighted images, b-value 0s/mm^2^, with one b0 volume acquired in the reversed phase encoded direction, acquisition time = 9 min 56 s) and resting-state functional MRI (rsfMRI) (multiband echo-planar imaging, TR = 1300 ms, TE = 40 ms, flip angle = 66°, field of view = 212 mm, voxel dimension = 2 mm isotropic, acquisition time = 10 min 10 s). For the rsfMRI scan subjects were instructed to lie in dimmed light with their eyes open, blink normally, but not to fall asleep.

### Neuroimaging data pre-processing

For neuroimaging data pre-processing we used standard FMRIB Software Library (FSL) tools v5.0 (http://fsl.fmrib.ox.ac.uk/fsl/fslwiki/) [21]. A detailed description of the data pre-processing steps can be found in [13]. The pre-processing pipeline for T1-weighted images included: re-orientating images to the standard (MNI) template, bias field correction, registration to the MNI template using both linear (FLIRT) and non-linear (FNIRT) registration tools and brain extraction. Brain tissues were segmented using FMRIB's Automated Segmentation Tool (FAST) that allows extracting measures of total grey matter (GM), white matter (WM) and cerebrospinal fluid (CSF) [22]. Brain tissues and sub-cortical regions were visually inspected to ensure an accurate segmentation, and manually edited where necessary.

The pre-processing pipeline for diffusion MRI data used a recently developed approach that simultaneously considers and corrects for susceptibility-induced distortions, eddy-currents and head motion, which is based on methods developed and applied to the Human Connectome Project (HCP) diffusion MRI data [23]. Fractional anisotropy (FA), mean diffusivity (MD), axial (DA) and radial diffusivity (DA) maps were generated using DTIFit, part of FMRIB’s Diffusion Toolbox, that fits a diffusion tensor model at each voxel [24].

Resting-state fMRI data pre-processing consisted of motion correction, brain extraction, high-pass temporal filtering with a cut-off of 100 s, and field-map correction and was carried out using FSL Multivariate Exploratory Linear Optimized Decomposition into Independent Components (MELODIC) [25]. To identify and regress out the “signal” of artefactual components reflecting non-neuronal fluctuations, we used single-subject independent component analysis (ICA) followed by an automatic component classification with FMRIB's ICA-based X-noiseifier (FIX) [26, 27]. The pre-processed and “cleaned” functional data was registered to the individual's structural scan and standard space images using FNIRT, then optimized using boundary-based-registration approach [28], and finally spatially smoothed using an isotropic Gaussian kernel of 6 mm full width at half maximum (FWHM).

### Neuroimaging data analysis

For data analysis we used standard FSL tools v5.0.

To study grey matter (GM) morphology, the T1-weighted structural imaging data were analysed using a voxel-based morphometry (VBM) approach. Whole-brain analysis was carried out with FSL-VBM [29], using default settings.

FMRIB’s Integrated Registration and Segmentation Tool (FIRST [30]), an automated model-based segmentation/registration tool, was applied to extract volumes of sub-cortical GM structures. Volumes were normalized to total intracranial volume and imported into SPSS for further statistical analyses.

White matter integrity (FA, MD, DA and DR) was analysed using FMRIB’s Diffusion Toolbox and tract-based spatial statistics (TBSS), a voxelwise approach for analysis of FA (and MD, DA, DR) data [31].

Functional connectivity was analysed using the FSL dual regression method [32]. Connectivity of 8 standard resting-state networks defined by a group ICA using MELODIC was analysed: the default-mode network, executive control network, two visual networks, the auditory and sensory-motor networks and left and right fronto-parietal networks [25].

For all image-based analyses (VBM, TBSS, dual regression) voxelwise general linear modelling (GLM) was applied using permutation nonparametric testing (5000 permutations) and P<0.05 correcting for multiple comparisons across space using threshold-free cluster enhancement (TFCE) [33]. Three *UCP*2 haplotype groups were compared in this model. Due to the significant effects of *UCP*2 polymorphisms on lifetime risk of mood disorders in men in this sample, we carried out two different analyses of the neuroimaging data. In the first analysis, all participants were included irrespective of sex and lifetime occurrence of mood disorder. One-way ANOVAs were performed to determine *UCP2* haplotype group effects on imaging measures and post-hoc tests were performed to compare means between the three groups. In the second analysis, we only included male participants and investigated main effects of *UCP2* haplotype and lifetime occurrence of mood disorders and their interaction on brain structure and function using a two-way ANOVA. Age was used as a covariate of no interest in all analyses.

### Non-image based statistics

We used SPSS software version 23 (SPSS Inc., Chicago, USA) for non-image-based statistical analyses and to compare volumes of subcortical structures. χ^2^ tests were used to compare allelic distributions and determine Hardy-Weinberg equilibrium as well as to compare categorical variables between the three *UCP*2 haplotype groups. Gene-dose effects were analyzed using Mantel-Haenszel linear-by-linear association χ^2^ tests. Linkage disequilibrium between the two *UCP*2 polymorphisms was determined using the online tool CubeX [34] (http://www.oege.org/software/cubex/). One-way ANOVAs and post-hoc tests with Bonferroni correction were used to compare continuous sociodemographic variables and subcortical volumes between haplotype groups. An alpha of P<0.05 was considered significant but adjusted for multiple comparison if necessary using Bonferroni correction (alpha was divided by the number of comparisons).

## Results

### Frequencies of *UCP*2 polymorphisms

Genotype data were available for 436 of the 563 individuals included in this analysis. Overall, the observed *UCP* genotype frequencies were in Hardy-Weinberg equilibrium (*p* = 0.62 for -866G>A and *p* = 0.57 for Ala55Val, Table 1) with values that are similar to those found in the 1000 genomes project (*p* = 0.59 for -866G>A and *p* = 0.58 for Ala55Val). The two polymorphisms showed strong linkage disequilibrium with D’ = 0.99 and r^2^ = 0.80.

**Table 1:** Comparisons of allelic distributions across the whole sample and between participants with and without mental health disorders.

	Whole sample				Male				Female			
	Total	MHD	No MHD	P	Total	MHD	No MHD	P	Total	MHD	No MHD	P
**n**	436	137	299		359	101	258		77	36	41	
**G(−866)A (rs659366)**				0.07				**0.013**				0.30
**G/G**	0.38	0.33	0.40		0.38	0.28	0.41		0.39	0.47	0.32	
**G/A**	0.48	0.48	0.48		0.48	0.51	0.47		0.48	0.39	0.56	
**A/A**	0.14	0.19	0.11		0.14	0.21	0.11		0.13	0.14	0.12	
**MAF (−866)A**	0.38	0.43	0.36		0.38	0.47	0.35		0.37	0.33	0.40	
**Ala55Val (rs660339)**				0.08				**0.007**				0.49
**C/C**	0.32	0.26	0.35		0.31	0.20	0.36		0.35	0.42	0.29	
**C/T**	0.51	0.53	0.50		0.52	0.56	0.50		0.48	0.42	0.54	
**T/T**	0.17	0.22	0.15		0.17	0.24	0.15		0.17	0.17	0.17	
**MAF 55Val**	0.43	0.48	0.40		0.43	0.52	0.40		0.41	0.38	0.44	

MAF: minor allele frequency, MHD: mental health disorder, P values are based on χ^2^ tests, significant P values are shown in bold.

### Effect of *UCP*2 polymorphisms on occurrence of neurodegenerative or mental health disorders

*UCP*2 polymorphisms did not significantly affect occurrence of neurodegenerative diseases in this sample. Occurrence of neurodegenerative disease was defined as having a clinical diagnosis of Alzheimer’s disease, Parkinsons’s disease, stroke, transient ischaemic attack, multiple sclerosis or mild cognitive impairment (based on clinical diagnosis or a MoCA score < 26). There was a non-significant trend to an overrepresentation of the minor alleles in participants with current or past diagnosis of mental health disorders, according to the SCID (χ^2^(2, n = 436) = 5.27, P<0.1 for -866A and χ^2^(2, n = 436) = 5.18, P<0.1 for 55Val) (Table 1). To study possible gender effects, we investigated male and female populations separately. The overrepresentation of both minor alleles was only significant in male participants (χ^2^_(2,436)_ = 8.67, P<0.05 for -866A and χ^2^(2, n = 436) = 9.94, P<0.01 for 55Val) but not in female participants (Table 1).

For the G(-866)A polymorphism, there was a higher risk of having current or past diagnosis of mental health disorder for men who carried one (OR = 1.63, 95% CI = 0.96–2.76) or two minor alleles (OR = 2.77, 95% CI = 1.38–5.57) compared with homozygotes for the major allele (Table 2). For the Ala55Val polymorphism there was a higher risk of having current or past diagnosis of mental health disorder for men who carried one (OR = 2.05, 95% CI = 1.15–3.64) or two minor alleles (OR = 2.91, 95% CI = 1.44–5.87) compared with homozygotes for the major allele (Table 2). The gene-dose effect was significant for both polymorphisms (χ^2^(1, n = 359) = 8.56, P<0.01 for -866A and χ^2^(1, n = 359) = 9.64, P<0.01 for 55Val).

**Table 2:** Comparisons of risk and odds between the genotypes in men.

	Total (n)	MHD (n)	No MHD (n)	RE	RR	OE	OR
**G(−866)A (rs659366)**							
**G/G**	135	28	107	0.21	1	0.26	1
**G/A**	174	52	122	0.30	1.44	0.43	1.63 (0.96–2.76)
**A/A**	50	21	29	0.42	2.03	0.72	2.77 (1.38–5.57)
**Ala55Val (rs660339)**							
**C/C**	112	20	92	0.18	1	0.22	1
**C/T**	185	57	128	0.31	1.73	0.45	2.05 (1.15–3.64)
**T/T**	62	24	38	0.39	2.17	0.63	2.91 (1.44–5.87)

MHD: mental health disorder, RE: risk estimates, RR: risk ratios (compared with homozygotes for major allele), OE: odds estimates, OR: odds ratios compared with homozygotes for major allele (with 95% confidence interval in brackets).

We then investigated whether specific diagnoses were overrepresented in those who carried one or two minor alleles of the two polymorphisms. 97 out of 101 participants who reported current or past episodes of mental health disorders suffered from mood disorders (defined as bipolar disorder, dysthymic disorder, recurrent or single episode major depressive disorder and unspecified mood disorders, which includes minor depression). The numbers for each of the disorders were insufficient to conduct any further statistical analyses.

We sought to replicate this effect in a larger Whitehall II sample of 5025 participants who took part in phase 9 (data collected from 2007–2009) and were genotyped for *UCP2*. However, there was no SCID data available for these participants. Therefore, participants were classified as having a current or past episode of mood disorder according to a definition used in previous Whitehall II studies [35]: a total score of 5 or more on the GHQ, a total score of 16 or more on the CES-D and the use of antidepressant medication in any of the previous Whitehall II phases. Using this metric, we were not able to replicate the significant effect of *UCP2* polymorphisms on occurrence of mood disorders in the larger sample.

### Neuroimaging data analysis of haplotype groups

Due to the strong linkage disequilibrium between the two *UCP2* polymorphisms, participants were grouped according to three different haplotype groups for all analyses of neuroimaging data: homozygotes for the major alleles (called haplotype group 1 (HT1)), homozygotes for minor alleles (HT3), and heterozygotes (HT2). Out of the 436 participants included in this analysis, 317 participants fell into one of these three groups and had complete neuroimaging datasets available. There were 112 participants in HT group 1, 155 in HT2 and 50 in HT3. These groups did not show any differences in socio-demographic variables (age, years of education, MOCA score, IQ or body-mass index (BMI), Table 3). Although the sex distribution in this sample is representative of the overall Whitehall II sample in that around 80% of all participants are male, the three haplotype groups did not significantly differ in sex distributions.

**Table 3:** Comparisons of socio-demographic variables between the haplotype groups included in the neuroimaging analysis.

	HT1 (GG/CC)	HT2 (GA/CT)	HT3 (AA/TT)	P Value
**n**	112	155	50	
**Age**	69.7 (5.4)	69.3 (5.4)	69.9 (5.5)	0.70
**Sex (n and % women)**	23 (20.5%)	25 (16.1%)	9 (18%)	0.65
**Total years of education**	15.7 (3.3)	15.6 (3.6)	15.4 (3.4)	0.88
**MOCA score**	27.4 (2.1)	27.4 (2.1)	27.3 (2.2)	0.93
**IQ**	118.9 (9.5)	118.7 (10.0)	117.9 (9.6)	0.82
**BMI**	25.6 (4.4)	26.6 (4.2)	26.8 (3.7)	0.1

Values denote mean (±Standard Deviation) or number of subjects, P-values refer to one-way ANOVAs (parametric data) and chi-square tests (categorical data).

Due to the significant effects of *UCP*2 polymorphisms on lifetime risk of mood disorders in men in this sample, we carried out two different analyses of the neuroimaging data. In the first analysis, all participants were included irrespective of sex and lifetime occurrence of mood disorder. There were no significant differences between *UCP2* haplotype groups for any of the neuroimaging measures investigated here. All non-thresholded statistical images for these analyses can be found on NeuroVault [36]: http://neurovault.org/collections/IAGLVJEJ/. In the second analysis, we only included male participants and investigated main effects of UCP2 haplotype group and lifetime occurrence of mood disorders and their interaction. Again, there was no significant main effect of *UCP2* haplotype group and no significant interaction between *UCP2* haplotype group and lifetime occurrence of mood disorders for any of the neuroimaging measures.

## Discussion

We set out to investigate possible effects of *UCP*2 polymorphisms on the risk of developing neurodegenerative disease but did not find significant effects in this sample. There are two possible explanations for this. On the one hand it is possible that the sample in this study was too small to detect significant effects and a larger study with more statistical power would be needed to rule this out. On the other hand it is possible that UCP2 polymorphisms do not play a role in the development of neurodegenerative disorders. This interpretation is backed up by the fact that *UCP2* polymorphisms have not been reported in genome-wide association studies (GWAS) that investigated genetic factors underlying risk of Alzheimer’s disease (e.g. [37]) or Parkinson’s disease (e.g. [38]). However, we observed a significant effect of *UCP2* polymorphisms on the occurrence of mood disorders in men in this sample. This effect was significant for both *UCP2* polymorphisms and showed a gene-dose effect in that those who carry two minor alleles had nearly threefold increased odds of lifetime occurrence of mood disorders and those with one minor allele a twofold elevated odds compared with those who are homozygote for the major alleles. While this is an intriguing finding, we could not replicate the effect in the larger Whitehall II sample. However, there are no data available for lifetime occurrence of mental health disorders for the larger sample, and we were only able to use proxy markers that will give an indication whether participants had a mood disorder when they were tested during the last 30 years. While *UCP2* polymorphisms have not been reported in GWAS for major depressive disorders (e.g. [39]) there are several reasons why it would still be worth following up on. First, patient data for GWAS often comes from those who have severe episodes of depression and are hospitalized. However, only very few participants included in this Whitehall II sub-study suffered from severe depression. In most cases they reported episodes of minor depression. Second, there are two important differences with regards to the age and sex distribution of the sample investigated here and those included in GWAS. The average age of participants in this study is around 70 years, which is different from many other samples that have a much lower average age, in most cases between 40 and 50 years (e.g. [40]). In addition, the sex distribution is different since around 80% of Whitehall II participants are men, whereas other samples have a much higher proportion of female patients from 50–80% (e.g. [40]). Therefore, *UCP2* polymorphisms might be genetic risk factors that affect lifetime occurrence of less severe mood disorders particularly in men. This would be a further indication of a link between mitochondrial (dys-)function and mood disorders [41]. However, it is a result that will need to be replicated independently.

To our knowledge this is the first study to investigate effects of *UCP2* polymorphisms on human brain structure and function using neuroimaging data. We did not find any significant effects of *UCP2* haplotype group on grey or white matter structure or resting-state functional connectivity. One interpretation is that *UCP2* polymorphisms do not affect brain structure or function. However, it is also possible that the sample investigated here was too small to find any significant effects, or that effects of *UCP2* polymorphisms on the brain are too subtle to be picked up by any of the neuroimaging measures here. Again, a larger study sample would be required to clarify these questions.

## Conclusion

In conclusion, we show in a sub-sample of the Whitehall II study that *UCP2* polymorphisms do not significantly affect grey or white matter structure or resting-state functional connectivity. An interesting effect of *UCP2* polymorphisms on lifetime occurrence of mood disorders requires independent replication.

## References

[1] Krauss S, Zhang CY, Lowell BB. The mitochondrial uncoupling-protein homologues. Nat Rev Mol Cell Biol. 2005;6(3):248–61. . 15738989 10.1038/nrm1592

[2] Diano S, Urbanski HF, Horvath B, Bechmann I, Kagiya A, Nemeth G, et al Mitochondrial uncoupling protein 2 (UCP2) in the nonhuman primate brain and pituitary. Endocrinology. 2000;141(11):4226–38. Epub 2000/11/23. doi: 10.1210/endo.141.11.7740 . 11089557 10.1210/endo.141.11.7740

[3] Nakase T, Yoshida Y, Nagata K. Amplified expression of uncoupling proteins in human brain ischemic lesions. Neuropathology: official journal of the Japanese Society of Neuropathology. 2007;27(5):442–7. . 18018477 10.1111/j.1440-1789.2007.00815.x

[4] Donadelli M, Dando I, Fiorini C, Palmieri M. UCP2, a mitochondrial protein regulated at multiple levels. Cell Mol Life Sci. 2014;71(7):1171–90. doi: 10.1007/s00018-013-1407-0 . 23807210 10.1007/s00018-013-1407-0 PMC11114077

[5] Cardoso S, Correia S, Carvalho C, Candeias E, Placido AI, Duarte AI, et al Perspectives on mitochondrial uncoupling proteins-mediated neuroprotection. J Bioenerg Biomembr. 2015;47(1–2):119–31. Epub 2014/09/15. doi: 10.1007/s10863-014-9580-x . 25217852 10.1007/s10863-014-9580-x

[6] Andrews ZB, Horvath B, Barnstable CJ, Elsworth J, Yang L, Beal MF, et al Uncoupling protein-2 is critical for nigral dopamine cell survival in a mouse model of Parkinson's disease. J Neurosci. 2005;25(1):184–91. doi: 10.1523/JNEUROSCI.4269-04.2005 . 15634780 10.1523/JNEUROSCI.4269-04.2005 PMC6725213

[7] Dietrich MO, Andrews ZB, Horvath TL. Exercise-induced synaptogenesis in the hippocampus is dependent on UCP2-regulated mitochondrial adaptation. J Neurosci. 2008;28(42):10766–71. doi: 10.1523/JNEUROSCI.2744-08.2008 ; 18923051 10.1523/JNEUROSCI.2744-08.2008 PMC3865437

[8] Dietrich MO, Horvath TL. The role of mitochondrial uncoupling proteins in lifespan. Pflugers Arch. 2010;459(2):269–75. doi: 10.1007/s00424-009-0729-0 ; 19760284 10.1007/s00424-009-0729-0 PMC2809791

[9] Andersen G, Dalgaard LT, Justesen JM, Anthonsen S, Nielsen T, Thorner LW, et al The frequent UCP2 -866G>A polymorphism protects against insulin resistance and is associated with obesity: a study of obesity and related metabolic traits among 17 636 Danes. Int J Obes (Lond). 2013;37(2):175–81. Epub 2012/02/22. doi: 10.1038/ijo.2012.22 . 22349573 10.1038/ijo.2012.22

[10] Qian L, Xu K, Xu X, Gu R, Liu X, Shan S, et al UCP2 -866G/A, Ala55Val and UCP3 -55C/T polymorphisms in association with obesity susceptibility—a meta-analysis study. PLoS One. 2013;8(4):e58939 doi: 10.1371/journal.pone.0058939 ; 23560041 10.1371/journal.pone.0058939 PMC3613358

[11] Rose G, Crocco P, De Rango F, Montesanto A, Passarino G. Further support to the uncoupling-to-survive theory: the genetic variation of human UCP genes is associated with longevity. PLoS One. 2011;6(12):e29650 doi: 10.1371/journal.pone.0029650 ; 22216339 10.1371/journal.pone.0029650 PMC3246500

[12] Marmot M, Brunner E. Cohort Profile: the Whitehall II study. Int J Epidemiol. 2005;34(2):251–6. Epub 2004/12/04. doi: 10.1093/ije/dyh372 . 15576467 10.1093/ije/dyh372

[13] Filippini N, Zsoldos E, Haapakoski R, Sexton CE, Mahmood A, Allan CL, et al Study protocol: The Whitehall II imaging sub-study. BMC Psychiatry. 2014;14:159 Epub 2014/06/03. doi: 10.1186/1471-244X-14-159 ; 24885374 10.1186/1471-244X-14-159 PMC4048583

[14] Goldberg D W P. A user’s guide to the general health questionnaire. London: GL Assessment Limited 2006.

[15] Nasreddine ZS, Phillips NA, Bedirian V, Charbonneau S, Whitehead V, Collin I, et al The Montreal Cognitive Assessment, MoCA: a brief screening tool for mild cognitive impairment. J Am Geriatr Soc. 2005;53(4):695–9. Epub 2005/04/09. doi: 10.1111/j.1532-5415.2005.53221.x . 15817019 10.1111/j.1532-5415.2005.53221.x

[16] Radloff L. The CES-D Scale: A Self-Report Depression Scale for Research in the General Population. Applied Psychological Measurement. 1977;1:385–401.

[17] Wechsler D. Test of Premorbid Functioning. UK Version (TOPF UK) Bloomington, MN: Pearson Inc; 2011.

[18] First M, Gibbon M, Spitzer R, Williams J. User's Guide for the Structured Clinical Interview for DSM-IV-TR Axis I Disorders—Research Version—(SCID-I for DSM-IV-TR), November 2002 Revision: New York: Biometric Research Department, New York State Psychiatric Intitute; 2002.

[19] Keating BJ, Tischfield S, Murray SS, Bhangale T, Price TS, Glessner JT, et al Concept, design and implementation of a cardiovascular gene-centric 50 k SNP array for large-scale genomic association studies. PLoS One. 2008;3(10):e3583 doi: 10.1371/journal.pone.0003583 ; 18974833 10.1371/journal.pone.0003583 PMC2571995

[20] van der Kouwe AJ, Benner T, Salat DH, Fischl B. Brain morphometry with multiecho MPRAGE. Neuroimage. 2008;40(2):559–69. doi: 10.1016/j.neuroimage.2007.12.025 ; 18242102 10.1016/j.neuroimage.2007.12.025 PMC2408694

[21] Smith SM, Jenkinson M, Woolrich MW, Beckmann CF, Behrens TE, Johansen-Berg H, et al Advances in functional and structural MR image analysis and implementation as FSL. Neuroimage. 2004;23 Suppl 1:S208–19. doi: 10.1016/j.neuroimage.2004.07.051 . 15501092 10.1016/j.neuroimage.2004.07.051

[22] Zhang Y, Brady M, Smith S. Segmentation of brain MR images through a hidden Markov random field model and the expectation-maximization algorithm. IEEE Trans Med Imaging. 2001;20(1):45–57. Epub 2001/04/11. doi: 10.1109/42.906424 . 11293691 10.1109/42.906424

[23] Sotiropoulos SN, Jbabdi S, Xu J, Andersson JL, Moeller S, Auerbach EJ, et al Advances in diffusion MRI acquisition and processing in the Human Connectome Project. Neuroimage. 2013;80:125–43. doi: 10.1016/j.neuroimage.2013.05.057 ; 23702418 10.1016/j.neuroimage.2013.05.057 PMC3720790

[24] Behrens T, Woolrich M, Jenkinson M, Johansen-Berg H, Nunes R, Clare S, et al Characterization and propagation of uncertainty in diffusion-weighted MR imaging. Magn Reson Med. 2003;50(5):1077–88. doi: 10.1002/mrm.10609 14587019 10.1002/mrm.10609

[25] Beckmann CF, DeLuca M, Devlin JT, Smith SM. Investigations into resting-state connectivity using independent component analysis. Philos Trans R Soc Lond B Biol Sci. 2005;360(1457):1001–13. Epub 2005/08/10. doi: 10.1098/rstb.2005.1634 ; 16087444 10.1098/rstb.2005.1634 PMC1854918

[26] Salimi-Khorshidi G, Douaud G, Beckmann CF, Glasser MF, Griffanti L, Smith SM. Automatic denoising of functional MRI data: combining independent component analysis and hierarchical fusion of classifiers. Neuroimage. 2014;90:449–68. doi: 10.1016/j.neuroimage.2013.11.046 ; 24389422 10.1016/j.neuroimage.2013.11.046 PMC4019210

[27] Griffanti L, Salimi-Khorshidi G, Beckmann CF, Auerbach EJ, Douaud G, Sexton CE, et al ICA-based artefact removal and accelerated fMRI acquisition for improved resting state network imaging. Neuroimage. 2014;95:232–47. doi: 10.1016/j.neuroimage.2014.03.034 ; 24657355 10.1016/j.neuroimage.2014.03.034 PMC4154346

[28] Greve DN, Fischl B. Accurate and robust brain image alignment using boundary-based registration. NeuroImage. 2009;48(1):63–72. Epub 2009/07/04. doi: 10.1016/j.neuroimage.2009.06.060 ; 19573611 10.1016/j.neuroimage.2009.06.060 PMC2733527

[29] Douaud G, Smith S, Jenkinson M, Behrens T, Johansen-Berg H, Vickers J, et al Anatomically related grey and white matter abnormalities in adolescent-onset schizophrenia. Brain. 2007;130(Pt 9):2375–86. Epub 2007/08/19. doi: 10.1093/brain/awm184 . 17698497 10.1093/brain/awm184

[30] Patenaude B, Smith SM, Kennedy DN, Jenkinson M. A Bayesian model of shape and appearance for subcortical brain segmentation. Neuroimage. 2011;56(3):907–22. doi: 10.1016/j.neuroimage.2011.02.046 ; 21352927 10.1016/j.neuroimage.2011.02.046 PMC3417233

[31] Smith SM, Jenkinson M, Johansen-Berg H, Rueckert D, Nichols TE, Mackay CE, et al Tract-based spatial statistics: voxelwise analysis of multi-subject diffusion data. Neuroimage. 2006;31(4):1487–505. Epub 2006/04/21. doi: 10.1016/j.neuroimage.2006.02.024 . 16624579 10.1016/j.neuroimage.2006.02.024

[32] Filippini N, MacIntosh BJ, Hough MG, Goodwin GM, Frisoni GB, Smith SM, et al Distinct patterns of brain activity in young carriers of the APOE-epsilon4 allele. Proc Natl Acad Sci USA. 2009;106(17):7209–14. doi: 10.1073/pnas.0811879106 . 19357304 10.1073/pnas.0811879106 PMC2678478

[33] Smith SM, Nichols TE. Threshold-free cluster enhancement: addressing problems of smoothing, threshold dependence and localisation in cluster inference. NeuroImage. 2009;44(1):83–98. Epub 2008/05/27. doi: 10.1016/j.neuroimage.2008.03.061 . 18501637 10.1016/j.neuroimage.2008.03.061

[34] Gaunt TR, Rodriguez S, Day IN. Cubic exact solutions for the estimation of pairwise haplotype frequencies: implications for linkage disequilibrium analyses and a web tool 'CubeX'. BMC bioinformatics. 2007;8:428 doi: 10.1186/1471-2105-8-428 ; 17980034 10.1186/1471-2105-8-428 PMC2180187

[35] Kivimaki M, Shipley MJ, Allan CL, Sexton CE, Jokela M, Virtanen M, et al Vascular risk status as a predictor of later-life depressive symptoms: a cohort study. Biol Psychiatry. 2012;72(4):324–30. doi: 10.1016/j.biopsych.2012.02.005 ; 22425413 10.1016/j.biopsych.2012.02.005 PMC3539141

[36] Gorgolewski KJ, Varoquaux G, Rivera G, Schwarz Y, Ghosh SS, Maumet C, et al NeuroVault.org: a web-based repository for collecting and sharing unthresholded statistical maps of the human brain. Frontiers in neuroinformatics. 2015;9:8 doi: 10.3389/fninf.2015.00008 ; 25914639 10.3389/fninf.2015.00008 PMC4392315

[37] Lambert JC, Ibrahim-Verbaas CA, Harold D, Naj AC, Sims R, Bellenguez C, et al Meta-analysis of 74,046 individuals identifies 11 new susceptibility loci for Alzheimer's disease. Nat Genet. 2013;45(12):1452–8. doi: 10.1038/ng.2802 ; 24162737 10.1038/ng.2802 PMC3896259

[38] Nalls MA, Pankratz N, Lill CM, Do CB, Hernandez DG, Saad M, et al Large-scale meta-analysis of genome-wide association data identifies six new risk loci for Parkinson's disease. Nat Genet. 2014;46(9):989–93. doi: 10.1038/ng.3043 ; 25064009 10.1038/ng.3043 PMC4146673

[39] Major Depressive Disorder Working Group of the Psychiatric GC, Ripke S, Wray NR, Lewis CM, Hamilton SP, Weissman MM, et al A mega-analysis of genome-wide association studies for major depressive disorder. Mol Psychiatry. 2013;18(4):497–511. doi: 10.1038/mp.2012.21 ; 22472876 10.1038/mp.2012.21 PMC3837431

[40] Wray NR, Pergadia ML, Blackwood DH, Penninx BW, Gordon SD, Nyholt DR, et al Genome-wide association study of major depressive disorder: new results, meta-analysis, and lessons learned. Mol Psychiatry. 2012;17(1):36–48. doi: 10.1038/mp.2010.109 ; 21042317 10.1038/mp.2010.109 PMC3252611

[41] Manji H, Kato T, Di Prospero NA, Ness S, Beal MF, Krams M, et al Impaired mitochondrial function in psychiatric disorders. Nat Rev Neurosci. 2012;13(5):293–307. doi: 10.1038/nrn3229 . 22510887 10.1038/nrn3229

